# Intra-arterial Chemotherapy in Patients With Metastatic or Locally Aggressive Pancreatic Adenocarcinoma: A Scoping Review

**DOI:** 10.7759/cureus.60696

**Published:** 2024-05-20

**Authors:** Mahi Basra, Hemangi Patel, Alejandro Biglione

**Affiliations:** 1 Osteopathic Medicine, Nova Southeastern University, Clearwater, USA; 2 Internal Medicine, Nova Southeastern University Dr. Kiran C. Patel College of Osteopathic Medicine, Fort Lauderdale, USA; 3 Internal Medicine, Wellington Regional Medical Center, Wellington, USA

**Keywords:** intra-arterial chemotherapy, flec regimen, regional chemotherapy, pancreatic adenocarcinoma, pancreatic cancer

## Abstract

Pancreatic adenocarcinoma refers to cancer of the pancreatic duct cells. It is normally diagnosed when it is at an advanced stage, making the prognosis poor. Systemic chemotherapy is the primary treatment approach for locally advanced or metastatic pancreatic cancer and has been shown to improve survival by eight to 16 weeks. However, it does not directly penetrate malignant tissue and has many side effects, such as hair loss, bone marrow suppression, and many gastrointestinal issues. A newer treatment modality, regional intra-arterial chemotherapy (IAC), focuses on targeting malignant tissue directly to improve survival and decrease systemic side effects. When IAC is used with gemcitabine (GEM) or FLEC (5-fluorouracil, leucovorin, epirubicin, and carboplatin), the response rate for advanced pancreatic cancer is significantly improved. This literature review introduces the use of hepatic intra-arterial chemotherapy in patients with metastatic pancreatic adenocarcinoma.

## Introduction and background

Pancreatic adenocarcinoma refers to cancer originating from pancreatic duct cells. The risk factors for developing pancreatic cancer include family history, obesity, type 2 diabetes, and tobacco use [1-3]. The staging of pancreatic cancer ranges from 0 to IV.

Stage 0 is when the cancer is confined to the top of the pancreatic duct cells, not spread outside of the pancreas. Stages IA and IB are when the cancer is confined to the pancreas and has not spread to lymph nodes. Stages IIA and IIB are when the cancer is confined to the pancreas, bigger than 4 cm, and may or may not spread to lymph nodes. Stage II is when the cancer has spread to nearby blood vessels or organs, but not distant sites. Stage IV is when the cancer spreads to distant organs, such as the liver, lungs, and distant lymph nodes. Stage V refers to metastatic spread [4,5]. 

Pancreatic cancer is divided into one of four categories, aside from staging, based on the spread of cancer: resectable, borderline resectable, locally advanced, and metastatic [1,2]. Localized pancreatic cancer is resectable, while locally advanced is unresectable [1]. Metastatic pancreatic cancer is defined as when the tumor has spread to distant sites such as the liver, lungs, and bone. The grading of cancer defines how aggressively the cancer will spread [4]. Locally advanced pancreatic cancer (APC) specifically has a five-year survival rate of 7% and a mean survival rate of nine to 12 months from the time of diagnosis, making it a fatal diagnosis [2,3]. 

The majority of patients with pancreatic cancer are not diagnosed until they are at the advanced stage (stage 3, T3 only) because early detection remains a challenge [2]. At the time of diagnosis, they are ineligible for surgical resection, which is the only known cure for pancreatic cancer [3]. 

In recent years, systemic chemotherapy has been used to treat stage 4 metastatic pancreatic cancer and has improved survival by eight to 16 weeks [2]. However, a major concern for systemic chemotherapy is its inability to penetrate the malignant tissue directly and the reduced absorption with systemic administration [2]. There are many known side effects of systemic chemotherapy, such as hair loss, bone marrow suppression, and gastrointestinal issues [6]. The most common systemic chemotherapy agent for pancreatic cancer is gemcitabine (GEM) [6]. Its response rate is known to be 5-15%, and it does not improve survival significantly, even when combined with other regimens [6]. 

Newer and more innovative treatment methods have been developed, aiming to enhance treatment accuracy by directly targeting malignant tissue to increase survival rates. Regional intra-arterial infusion chemotherapy (AIC) helps to deliver high concentrations of chemotherapy directly into the malignant tissue and decreases the systemic side effects as seen in systemic chemotherapy [2,3]. Intra-arterial chemotherapy (IAC) with GEM specifically has been shown to improve response rates in advanced pancreatic cancer (APC), which otherwise has an overall survival (OS) of less than six months [6]. Another common IAC regimen, which shows potential for improving survival rates, is FLEC (5-fluorouracil, leucovorin, epirubicin, and carboplatin), which shows potential in improving survival rates. This literature review aims to introduce the use of intra-arterial chemotherapy in patients with metastatic and locally advanced pancreatic adenocarcinoma. 

## Review

Materials and methods 

Search Strategy and Selection Criteria 

A literature search was performed on January 15, 2024, utilizing the EMBASE, Medline ProQuest, and PubMed databases. This systematic review utilized the Preferred Reporting Items for Systematic Reviews and Meta-Analyses (PRISMA) (AP1) statement by the Cochrane Collaboration. Either clinical trials, cohort studies, cross-sectional trials, or primary studies should have been conducted between January 1st, 2014 and January 1st, 2024. Review articles, studies performed in patients with resectable and borderline resectable cancer, and non-English articles were excluded. Additionally, articles were excluded if they did not utilize intra-arterial chemotherapy. These criteria were selected to fulfil the objectives of this review. 

Key Terms

The key terms used to search for articles were: intra arterial chemotherapy, arterial chemotherapy, hepatic chemotherapy, pancreatic cancer, locally advanced pancreatic cancer, metastatic pancreatic cancer, and pancreatic metastases. Databases were searched using the Boolean operators “AND” and “OR” as follows: ((“intra-arterial chemotherapy*” OR (“intraarterial chemotherapy*” OR “arterial chemotherapy*” OR “hepatic intra-arterial*” OR “hepatic chemotherapy*” OR “hepatic intraarterial*”)) AND ( "pancreatic adenocarcinoma," " neuroendocrine pancreatic cancer," "exocrine pancreatic cancer," “pancreatic cancer*” OR “metastatic pancreatic cancer*” OR “metastatic*” OR “pancreatic metastases*")). Articles were further screened to include articles based on the aforementioned inclusion criteria.

Evaluation Process 

**Figure 1:**
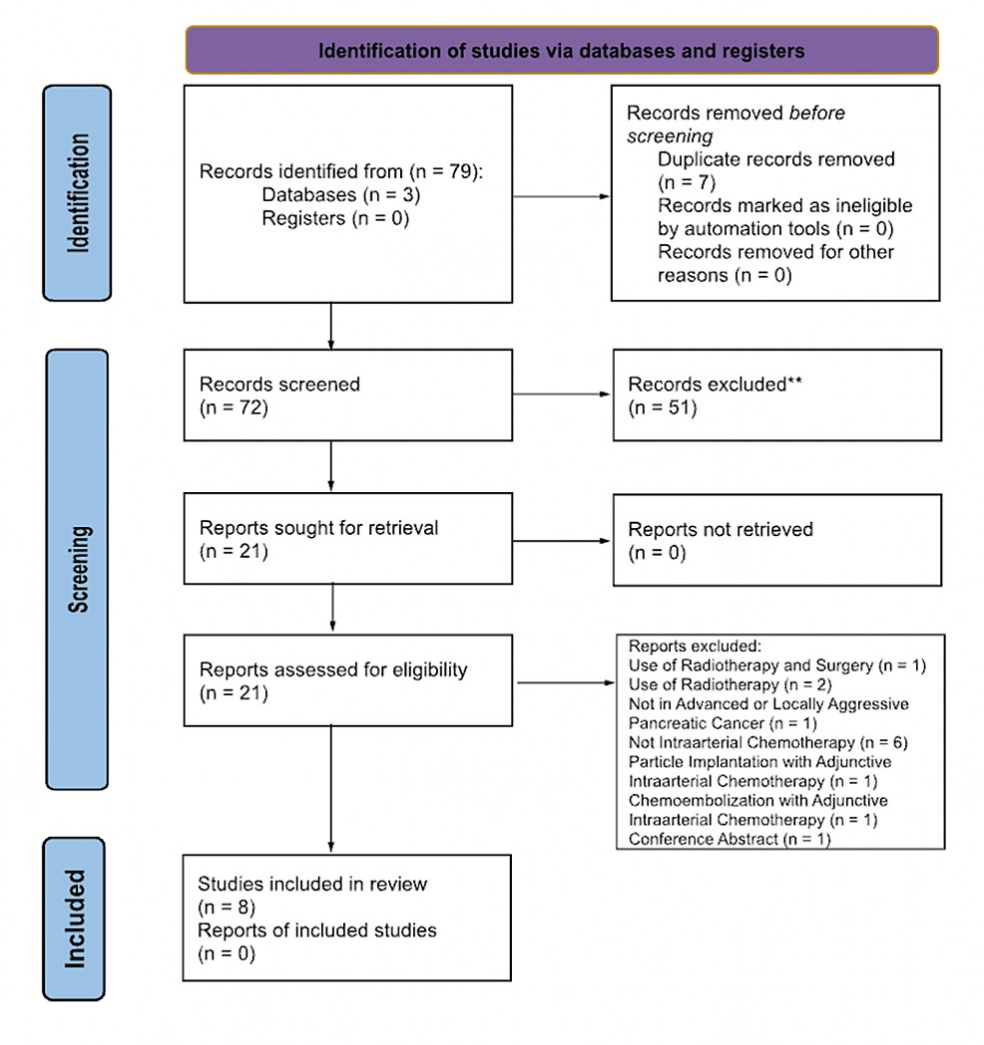
PRISMA diagram. *The number of records identified from each database or register searched. **Indicates how many records were excluded by a human, not automation tools. PRISMA: Preferred Reporting Items for Systematic Reviews and Meta-Analyses.

The process of inclusion for the articles is portrayed in the Preferred Reporting Items for Systematic Reviews and Meta-Analyses (PRISMA) chart (Figure 1). Seventy-nine total articles were obtained. The authors (MB and HP) evaluated 72 articles after seven duplicates were removed. Based on inclusion and exclusion criteria, 51 articles were excluded and 21 were sought for retrieval. Twenty-one were obtained and assessed for eligibility. The authors (MB and HP) each independently reviewed the full-text publications. After further analysis, two articles were excluded due to the use of radiation therapy, one was excluded due to the use of radiotherapy and surgery, one was excluded because it was not about patients with metastatic or locally advanced pancreatic cancer, six were excluded due to absence of intra-arterial chemotherapy treatment, one was excluded due to adjunctive particle implantation, one was excluded due to adjunctive chemoembolization, and one article was excluded because it was a conference abstract. Disagreements were resolved by discussion with all the aforementioned authors, yielding eight total studies included in the review. No additional references were utilized. 

Data Collection 

Each of the eight articles selected was qualitatively evaluated, and a results table was established with the following columns, as seen in Table 1: author, publication year, study design, sample size, outcomes, study limitations, and future recommendations. The review was written by the authors after the data was fully analyzed. 

**Table 1:** Results table. IA: intra-arterial, RIAC: regional intra-arterial chemotherapy, FLEC: 5-fluorouracil, leucovorin, epirubicin and carboplatin, ECOG: Eastern Cooperative Oncology Group, RCT: randomized control trial.

Reference	Study type	Sample size	Aim	Findings	Limitations	Recommendations
Liu et al., 2016 [2]	Retrospective study	Fifty-four patients with advanced unresectable pancreatic adenocarcinoma	Investigate prognostic factors in patients who had IA for advanced pancreatic cancer	Gemcitabine-based regional IA chemo presented a potential treatment method for advanced pancreatic adenocarcinoma	Only patients with RIAC included, limited to ethnic background	Increase sample size, diverse patient population selection, and clinical trials are further needed to assess the effects of RIAC to systemic chemotherapy and focus on adverse effects
Milandri et al., 2007 [7]	Prospective study	Nineteen chemotherapy-naive patients with measurable lesions	Determine response rates and time to progression in patients with locally advanced or metastatic pancreatic cancer 944	25% of patients had partial response, 44% had stable disease, and 31% had progressive disease. Median overall survival was six months	Small sample size	Repeat in larger sample size
Mambrini et al., 2008 [8]	Prospective study	Two hundred and eleven patients with advanced pancreatic cancer who underwent FLEC regimen	Identify prognostic factors of patients who underwent the same regiment of IA chemotherapy	Median overall survival was 9.2 months	Only used one regimen	Repeat to detect survival differences
Mori et al., 2020 [9]	Prospective study	Forty-five patients	Assess arterial administration of DNA crosslinking agents after intravenous administration of low-dose gemcitabine for patients with advanced pancreatic cancer	Can be a new treatment for locally advanced pancreatic cancer	CT angiography is needed for interventional radiologists, only for locally advanced pancreatic cancer not metastatic with advanced pancreatic cancer, no control study of IV low-dose gemcitabine, small sample size, no RCT	Bigger sample size, randomized control trial, control study group of IV low-dose gemcitabine to compare
Mambrini et al., 2009 [10]	Phase I study	Fifteen patients with locally advanced, surgically unresectable or metastatic pancreatic adenocarcinoma	Combination of IA chemo and systemic chemo	Limited toxicity found	Small sample size	Following doses are suggested: on day one every 28 days intra-arterial epirubicin 35 mg/m^2^ and cisplatin 42 mg/m^2^, on day two systemic gemcitabine at 1,000 mg/m^2^ and on days two to 15 capecitabine at 650 mg/m^2^ twice a day
Sawaski et al., 2022 [11]	Retrospective study	Eighty-one patients with ECOG performance status of 0-1 were enrolled; administered meds mentioned in abstract aim: analyze the efficacy and toxicity of these treatments	Analyze the efficacy and toxicity of these treatments	Metastatic sites included liver, peritoneum, distant lymph nodes, and lungs; within 28 days of initiation, 15 patients had common greater than or equal to 3 grade hematological adverse events; the major reason of the discontinuation was peripheral neuropathy	Small sample size in those who actually participated in a study	Increase sample size
Beane et al., 2015 [12]	Phase I clinical trial	Six patients with biopsy confirming borderline or unresectable pancreatic adenocarcinoma patients treated with increasing doses of gemcitabine administration	Determine the toxicity of regional intra-arterial gemcitabine delivered as a 24-h continuous infusion to the pancreas	Catheter placement and gemcitabine infusion were successful in all six patients; four doses were received; two patients developed grade 3 and grade 4 duodenal ischemia and upper GI bleeding; median overall survival was 15.3 months and median time to progression was three months; three patients progressed systematically; two patients had stable disease greater than four months after treatment and underwent pancreaticoduodenectomy	Small sample size	Repeat in a larger population

Results

FLEC Regimen

Milandri et al. conducted a study to understand the response rate and time to progression in patients with locally advanced or metastatic pancreatic cancer who were treated with intra-arterial chemotherapy. Nineteen patients who never had chemotherapy were treated with 5-fluorouracil 1000 mg/m^2^, leucovorin 100 mg/m^2^, epirubicin 60 mg/m^2^ (FLEC), and carboplatin 300 mg/m^2^, intra-arterially every 21 days. The FLEC regimen is a four-drug chemotherapy regimen that was shown to improve survival in patients with metastatic pancreatic cancer. On days four to 10 of the cycle, granulocyte colony-stimulating factors were administered prophylactically. Out of the 19 patients, 16 were available for response. About 25% of patients (4) had a partial response, 44% maintained stable disease (7), and 31% developed further disease (5). The response rate of the marker was 43%. Time to progression was four months on average, and overall survival (OS) length was six months. No grade 4 toxicity or severe complications were observed in association with the angiographic procedure [7]. 

Two hundred eleven patients who were assessed on their prognostic factors relative to pancreatic cancer. All patients received the FLEC regimen every three weeks via the celiac axis. One-hundred and eighty-two out of 211 patients complained of pain, with a median value of 40 mm on the visual analog scale. Age, tumor site, tumor size, grading, baseline pain, sex, and carbohydrate antigen (CA)-19-9 at baseline and after IAC had no significant survival influence. Patients with locally advanced disease had a median survival of 10.5 months when compared to those with metastatic disease, whose median survival was 6.6 months (p = 0.002). Patients who had less than or equal to three cycles of IAC had 5.9 months of median survival. Compared to 12.3 months of patients who underwent more than three cycles, this is statistically significant (p = 0.0001). Mambrini et al. also concluded that the FLEC regimen was well tolerated. Only one iliac artery intima dissection related to the angiographic procedure was observed out of 764 cycles. Twenty percent of patients had grade III to grade IV thrombocytopenia; two percent of patients had hematemesis during the thrombocytopenic period; 19% of patients had leukopenia; and 14% had anemia. Other reported effects were nausea, vomiting, and alopecia [8]. 

Combination Chemotherapy With Gemcitabine 

Renovocath (RC-120) is a dual-balloon catheter that is introduced through the femoral artery, allowing for drug infusion through a port located between both balloons. Rosemurgy et al. performed the first human study to evaluate the safety of the RC-120 catheter in patients with locally advanced pancreatic cancer. There were 20 patients with stage 3 locally advanced unresectable cancer were enrolled in the study. Each patient underwent a 28-day cycle of two doses of gemcitabine on day one and day 15 of each cycle. Over four cycles, doses of gemcitabine were increased from 250 mg/m^2^ to 500 mg/m^2^ to 750 mg/m^2^ to 1000 mg/m^2^. Doses were escalated if no dose-limiting toxicities occurred. The RC-120 device was introduced to the celiac, splenic, common hepatic, gastroduodenal, and superior mesenteric arteries based on each tumor location [9]. 

Increased expression of Rad51 in recombination repair of a DNA double-stranded break has been shown in pancreatic cancer. Thus, pretreatment with a Rad-51 inhibitor followed by arterial chemotherapy may be beneficial for patients. Gemcitabine is a Rad-51 inhibitor that may help in increasing drug concentration at tumor sites and limit gastrointestinal complications [9]. 

Mori et al. conducted a single-center prospective study aiming to investigate the efficacy of arterial administration of DNA crosslinking agents with low-dose intravenous gemcitabine. Forty-five patients with advanced, unresectable pancreatic ductal adenocarcinoma were enrolled in the study. Twenty-three patients had arterial or portal vein invasion in the locally advanced group, while 22 patients in the metastatic group had hepatic or distal nodal metastasis. All patients underwent three weeks of intravenous weekly gemcitabine administration and arterial administration of mitomycin C and epirubicin hydrochloride at the fifth or sixth week. Dosages of 150-500 mg/m^2^ gemcitabine were administered weekly on days one, eight, and 15. Patients began with 500 mg/m^2^ and decreased accordingly depending on each patient’s bone marrow suppression. Then, 30 mg of epirubicin hydrochloride and 15 mg of mitomycin C were diluted in saline and contrast agent and were administered via microcatheter at the origin of each supplying artery. This was repeated at 1.5 to two-month intervals until pancreatic mass was not present on imaging or until severe adverse effects were noted or if the patient wished to discontinue. All patients underwent at least one treatment course. Thirty-eight patients received at least two treatment courses. Overall 182 treatment courses were administered. The median OS time with locally advanced cancer was 23 months (95% confidence interval (CI) = 15.4-40.3 months). These patients (10 patients) with excellent treatment compliance had OS of 33 months (95% CI = 16.8-74.3 months), while those with poor treatment compliance (13 patients) had 17 months (95% CI = 11.0-21.8). In the metastatic cancer patient group, the median OS was 13 months (95% CI = 10.2-17.8 months). Those patients (10 patients) with excellent treatment compliance had OS of 17 months (95% CI = 11.6-25.2 months), while patients with poor treatment compliance (12 patients) had OS of eight months (95% CI = 5.9-15.0 months). Median local progression-free survival (LPFS) was 17.5 months in the locally advanced cancer group (95% CI = 10.1-36.0 months), while LPFS in the metastatic advanced group was eight months (95% CI = 6.2-14.5 months). Tumor response was achieved in 80.4% of patients. Of the patients, 69.6% complained of mild nausea and fatigue, 13% of patients had severe bone marrow suppression (anemia, leukopenia, and thrombocytopenia), 6.5% of patients had interstitial pneumonitis, and 6.5% had hemolytic uremic syndrome, and 2.2% had severe vomiting [9]. 

Mambrini et al. aimed to identify the adequate dose of gemcitabine in conjunction with capecitabine and intra-arterial cisplatin and epirubicin with 15 patients in the trial. Eligibility criteria included, the patient must: have disease in progression after at least one line of chemotherapy or chemotherapy/radiotherapy; histologically proven, surgically unresectable locally advanced or metastatic pancreatic adenocarcinoma; 18-80 years of age and adequate organ function. 35 mg/m^2^ epirubicin and 42 mg/m^2^ cisplatin were given on day one through the celiac axis via the Seldinger method in the femoral artery. 650 mg/m^2^ gemcitabine was given on day two of each cycle at 1,000 mg/m^2^ with a 30% dose increase for each consecutive patient cohort. The three patients who received gemcitabine 1000 mg/m^2^ on day two tolerated the treatment well. In the three patients who received 1300 mg/m^2^, grade 3 thrombocytopenia was observed. Another six patients were treated with 1300 mg/m^2^, two patients had grade 4 thrombocytopenia, and one patient had grade 4 neutropenia. The last three patients, treated with 1000 mg/m^2^ gemcitabine did not have any toxicity. Thus, the dose-limiting toxicity was myelotoxicity occurring at 1300 mg/m^2^ of gemcitabine [10]. 

A single-center retrospective study was performed by Sawasaki et al. to identify the efficacy and toxicity of intra-arterial gemcitabine, nab-paclitaxel, oxaliplatin, and itraconazole (GnPO-ITC). There were 81 patients with an Eastern Cooperative Oncology Group (ECOG) status of 0-1 who were administered 125 mg/m^2^ nab-paclitaxel, 1000 mg/m^2^ gemcitabine, 85 mg/m^2^ oxaliplatin on day one, and 400 mg itraconazole on day two. This cycle is repeated every two weeks. After 28 days of chemotherapy, 19% of patients reported greater than or equal to grade 3 hematological adverse effects. About 44% reported peripheral sensory neuropathy. The treatment overall response rate was 64% (95% CI = 54-75%). Median OS was 14.4 months (95% CI = 11.4-17.3 months). Median OS was 14.4 months (95% CI = 11.4-17.3 months). Median progression-free survival was 8.3 months (95% CI = 6.8-9.8 months). Eighty-eight of the patients required second-line treatment with irinotecan. Thus, concluding that the GnPO-ITC regimen had promising results in improving OS with acceptable toxicities [11].

There were 354 patients with advanced pancreatic cancer without any prior treatment who were recruited to investigate prognostic factors in patients who were treated with gemcitabine IAC. Seventy-two patients had distant metastases. 1000 mg/m^2^ of gemcitabine, followed by 100 mg/m^2^ of oxaliplatin, were delivered. In patients with lesions in the pancreatic head, 1/3 of the drug was delivered via the superior mesenteric artery, while 2/3 was delivered via the gastroduodenal artery. In patients with pancreatic body or tail lesions, the drug was delivered either via the great pancreatic artery, caudal pancreatic artery, or dorsal pancreatic artery. Two hundred seventy-four patients completed one cycle of intra-arterial infusion, while 80 patients completed two or more cycles. Median OS for all 354 patients was seven months (95% CI = 6-8 months). Those who received two cycles had a median OS of seven months (95% CI: 6-9 months), while the patient group who received one cycle had a median OS of six months (95% CI: 5-8 months). Results showed that patients who received regional intra-arterial chemotherapy (RAI) at a younger age had a pretreatment CA-19-9 value of less than 1000 U/mL, and tumor location at the head of the pancreas had a better outcome. This was determined by a decline in the CA-19-9 value following intra-arterial infusion [2].

While gemcitabine therapy has been shown to improve one-year survival for patients with advanced pancreatic ductal adenocarcinoma, dose-limiting toxicities are present. A more favorable toxicity profile has been noted in literature when administered at smaller doses over a long period of time when compared to a standard dosing regimen. Beane et al. aimed to establish the feasibility and toxicity of gemcitabine regional intra-arterial infusion delivered over 24 hours, continuously. Six patients with borderline or unresectable pancreatic adenocarcinoma who had been treated with at least one line of chemotherapy underwent vascular redistribution to the head of the pancreas. This was accomplished via arterial coil embolization, followed by perfusion catheter placement within the splenic artery. Gemcitabine was administered in three cohorts: 18 mg/m^2^/24 hours (cohort 1), 36 mg/m^2^/24 hours (cohort 2), and 72 mg/m^2^/24 hours (cohort 3) [12]. 

A subclavian approach for placement of perfusion catheter due to migration or celiac axis encasement was required in two out of six patients and four out of six patients required port infusion revision due to infusion catheter migration. One patient required three revisions of the port site due to port dislocation and catheter migration. A total of 33 cycles were administered via dose escalation. The median number of cycles administered was four per patient (range: 4-9 cycles). No toxicities were noted in patients treated with 18 mg/m^2^ dose. The one patient treated with the 115 mg/m^2^ dose developed anemia, duodenal ulcer, and gastroparesis. Four patients developed a grade 3 of higher adverse events ranging from nausea and fatigue to anemia to duodenal ulcer and gastroparesis and two patients presented with melena secondary to upper gastrointestinal bleeding and anemia requiring multiple blood transfusions. In the first patient, this occurred at the 36 mg/m^2^ dose on cycle four of gemcitabine administration. In the second patient, melena presented two weeks after infusion at 115 mg/m^2^ dose. Both patients recovered with proton pump inhibitor therapy and multiple transfusions delivered intravenously [12]. The median OS was 15.3 months (range: 3-17 months). The median time to progression was two months (range: 1-22 months) [12]. Table 1 shows the results of all the articles. 

Discussion

The FLEC regimen (5-fluorouracil 1000 mg/m^2^, leucovorin 100 mg/m^2^, epirubicin 60 mg/m^2^ (FLEC), and carboplatin 300 mg/m^2^) is a chemotherapy regimen that is administered intra-arterially and shows potential in improving survival in patients with metastatic pancreatic cancer [7]. When FLEC was administered via the celiac axis, the average survival for locally advanced disease was 10.5 months on average, while for metastatic cancer, it was 6.6 months [8]. Notably, the FLEC regimen suggests potential for intra-arterial chemotherapy; however, has side effects such as hair loss, gastrointestinal dysfunction, and bone marrow suppression [8]. 

An alternative treatment approach involves the use of Renovocath (RC-120), a dual-balloon catheter introduced through the femoral, celiac, splenic, common hepatic, gastroduodenal, and superior mesenteric arteries depending on the tumor's location [9]. When treated with Gemcitabine (GEM), a Rad-51 inhibitor, there are better outcomes because of the presence of higher levels of chemotherapy at tumor sites, thereby reducing gastrointestinal side effects [9]. When GEM was administered with intra-arterial chemotherapy, the overall survival in patients with excellent treatment compliance was 33 months, while in patients with poor treatment compliance, it was 17 months [9]. Metastatic cancer patients with poor treatment compliance had an overall survival of eight months, compared to 17 months for those with excellent treatment compliance, resulting in a median overall survival of 13 months [9]. The average progression-free survival was 17.5 months for locally advanced patients and eight months for metastatic patients. 

Adverse effects are inherent and present in all chemotherapy regimens. In the patients treated with GEM and intra-arterial chemotherapy, 69.6% experienced nausea and fatigue, while less than 13% of patients experienced either thrombocytopenia, leukopenia, or anemia, highlighting the reduced adverse symptoms when treated with GEM plus intra-arterial chemotherapy [9]. The dose-limiting toxicity was identified as myelotoxicity at GEM 1300 mg/m^2^. 

Another intra-arterial chemotherapy regimen is gemcitabine, nab-paclitaxel, oxaliplatin, and itraconazole (GnPO-ITC). GnPO-ITC yielded an overall survival rate of 14.4 months and progression-free survival of 8.3 months [11]. The primary toxicity observed was peripheral sensory neuropathy in 44% of patients [11].

A consideration for GEM therapy involves determining the appropriate dose to prevent toxicity. Patients treated with GEM 18 mg/m^2^ experienced no toxicities, while higher doses (36 mg/m^2^ and 115 mg/m^2^) led to adverse effects such as anemia, duodenal ulcers, gastroparesis, and upper GI bleeds [12]. The presence of adverse effects at higher doses should be taken into consideration when treating patients. Close monitoring is crucial to minimize toxicity in patients undergoing treatment.

Limitations

For this study, it is critical to consider the limitations of evaluating medical literature and the interpretation of results. In this review, some primary studies may have been missed since only three databases were analyzed and articles in other languages than English were excluded. Another common limitation seen in these studies was small sample sizes and lacking diverse patient populations, making it difficult to extrapolate these results to a larger, general population. Additionally, earlier studies may have been missed due to the 2014 cutoff. However, this is a relatively new concept and technique and thus may be minimal. 

Future Studies

Future studies should be conducted in a larger patient population to note adverse effects and effective therapeutic regimens/dosages. Additionally, studies should be repeated in a more diverse patient population. Researchers may also investigate the correlation between varying drug concentrations at the site and their corresponding adverse effects to understand the precise relationship between dosage and side effects. Future studies focused on particle implantation in combination with intra-arterial chemotherapy and chemoembolization may also be useful. 

## Conclusions

This literature review aims to analyze the use, outcomes, benefits, and adverse effects of intra-arterial chemotherapy in patients with locally advanced and metastatic pancreatic adenocarcinoma. Locally advanced pancreatic cancer is unresectable and generally has vast vascular involvement, while metastatic cancer spreads to other organs. Intra-arterial chemotherapy poses a viable adjunct to treatment, directly delivering pharmaceuticals to the malignant tissue. Studies performed showed that the FLEC regimen may have overall decreased side effects and may increase overall survival up to 10.5 months. GEM treatment is an additional treatment regimen that showed promising results. Overall, future studies are needed to explore the role of interventional oncology in the treatment of advanced pancreatic adenocarcinoma. 
